# A Novel Six-Point Supraperiosteum Injection with Calcium Hydroxyapatite for Jawline Refining and Facial Anti-aging in Asian Patients

**DOI:** 10.1007/s00266-025-05045-x

**Published:** 2025-07-03

**Authors:** Bing-Qi Wu, Yun-Jhen Lin, Chang-Cheng Chang, Man-Lok Lio, Wei-Chun Chang, Hsiu-Mei Chiang, Yung-Hsueh Huang, Ying-Chuan Hsu

**Affiliations:** 1 Department of Education, China Medical University Hospital, Taichung, 404 Taiwan; 2 School of Medicine, College of Medicine, China Medical University, No. 2, Yude Rd., North Dist., Taichung, 40402 Taiwan; 3 Department of Medical Education, Kaohsiung Chang Gung Memorial Hospital, Kaohsiung, 833 Taiwan; 4 Department of Cosmeceutics, China Medical University, Taichung, 40402 Taiwan; 5 Division of Plastic and Reconstructive Surgery, Department of Surgery, China Medical University Hospital, Taichung, 404 Taiwan; 6 Aesthetic Medical Center, China Medical University Hospital, Taichung, Taiwan; 7 Department of Obstetrics and Gynecology, China Medical University Hospital, Taichung, 404 Taiwan; 8 20skin Four Seasons Clinic, Taichung, 408 Taiwan

**Keywords:** Calcium hydroxylapatite, Anti-aging, Supraperiosteal injection, Facial rejuvenation

## Abstract

**Background:**

Supraperiosteal injection by calcium hydroxyapatite (CaHA) fillers is an efficient treatment for facial rejuvenation to achieve bony recontouring, facial augmentation and lifting. However, a safe and efficient injection technique for Asians was not described, and an assessment scale to quantify “facial anti-aging” does not exist.

**Methods:**

We enrolled 16 Asian women (*n* = 32) to receive a single session of six-point calcium hydroxyapatite (CaHA, Radiesse® Plus) filler injections between July 1, 2021, and July 1, 2022. The six injection sites included the eyebrow arch, lateral eyebrow, lateral zygoma, piriform fossa, lateral chin, and anteromedial cheek. Efficacy was assessed at weeks 0 (immediately post-treatment), 4, 12, and 24 using a specialized angular and length measurement system, with outcomes further evaluated using an 11-level anti-aging scale.

**Results:**

Among the four angular and six linear parameters assessed, statistically significant variations were observed across five time points. Regarding post hoc analyses, the Eyebrow-Tail angle showed a statistically significant change at week 24, increasing by 2.84° (*p* < 0.001) and corresponding to an anti-aging scale value of 6. This parameter indicated eyebrow lifting. The Tragus-Oral (−10.4 mm, *p* = 0.004) and Lower Facial Contouring (−9.8 mm, *p* = 0.044) showed statistically significant reductions and received aging scale ratings of 10 and 9, respectively, at week 24. These two parameters indicated achieving jawline refining. Overall, the final anti-aging scale improved from an average of 50–59, with minimal or ignorable ecchymosis and swelling at week 24.

**Conclusions:**

The 6-point calcium hydroxyapatite filler injection achieved facial rejuvenation. After a single session, the injection recontoured the jawline and lifted the eyebrow tail.

**Level of Evidence IV:**

This journal requires that authors assign a level of evidence to each article. For a full description of these Evidence-Based Medicine Ratings, please refer to Table of Contents or online Instructions to Authors www.springer.com/00266

**Supplementary Information:**

The online version contains supplementary material available at 10.1007/s00266-025-05045-x.

## Introduction

Facial aging is a complex, interrelated process involving changes to the bone, fat compartment, muscle and skin [1]. Distinct from the earlier concept of sagging effects by gravity to the overlying skin and superficial musculoaponeurotic system (SMAS), current concept has been proposed that the loss of volume from fundamental layers also plays an important role [2, 3]. These losses of volume, particularly due to bone resorption and loss of adjacent fat volume from different facial compartments, leads to deflation, flattening and pseudoptosis of the soft tissues. In this case, “revolumnizing” or “restoring” the loss tissue via soft-tissue filler is a powerful and optimal treatment to achieve facial rejuvenation from inside-out.

Radiesse®(+) (Merz Pharma GmbH & Co. KGaA) is a biostimulatory filler containing 30% synthetic CaHA suspended in a 70% aqueous sodium carboxymethylcellulose gel matrix [4]. Once the material was injected, the carrier carboxymethylcellulose gel initiates the augmentative effect, but after a period of resorption time (approximately 9–12 months), the 25- to 45-μm CaHA microspheres contact directly with tissue to trigger collagenesis and sustain volumization [4–6]. Unlike the previous version, the pain control and non-migrating effects of the integrated filler allow it as an ideal option for supraperiosteally bold-lifting. However, the common technique of injection was generally in the dermal region and a standardized technique for Asians is undefined. We modified the injecting points into six landmarks to conclude the whole-face safely and effectively.

Assessing the efficacy of different facial rejuvenative procedures is complex with various subjective perceptions, leading to an obstacle for communicating between patients and physicians. We had designed a specific assessment system with four angular and six linear measurements between static and mobile points to assess subtle changes of different facial aesthetic procedures, results and effectiveness were both validated [7–10]. The score from each ten measurements was further integrated into an anti-aging scale ranging from 0 to 100. The total score for the entire face is 100. Scores lower or higher than 50 suggest “aging or rejuvenation, respectively.”

Our study introduced a single session of calcium hydroxyapatite fillers treatment via 6-point supraperiosteal injection to achieve facial rejuvenation. We evaluated the efficacy by using our new integrated anti-aging scale to obtain comprehensive and formative results.

## Materials and Methods

### Ethical Statement

All participants provided written informed consent for the trial, including consent for publication, and all procedures were conducted following the Declaration of Helsinki.

### Study Sample

Sixteen Asian female participants between the ages of 20 and 60 years were enrolled in our prospective clinical review. All participants provided written informed consent for the trial. They undergo one treatment of modified bold-lift 6-point calcium hydroxyapatite fillers injection (CaHA, Radiesse®) between July 1, 2021, and July 1, 2022. Individuals were excluded if they had undergone plastic surgery (e.g., facelift, facial resurfacing, or blepharoplasty) or any cosmetic procedures before or between the aforementioned dates; allergic to dermal fillers, pregnant, or did not consent to the publication of their images.

### Procedures and Devices

**Fig. 1:**
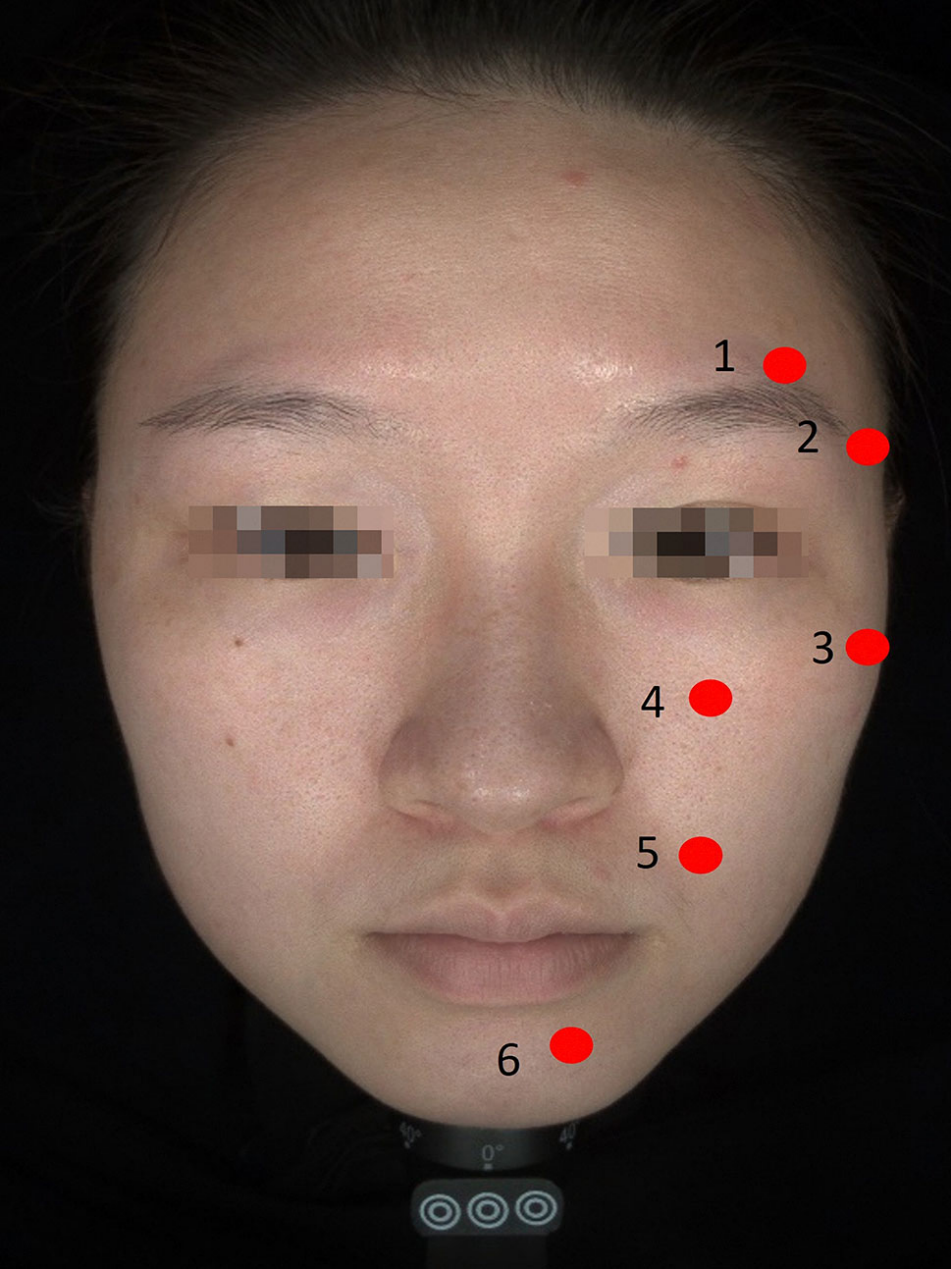
The illustration of our six-point calcium hydroxyapatite injection (presented on left side; same points on right side). 1. Eyebrow arch; 2. Lateral eyebrow; 3. Lateral zygoma; 4. Anteromedial cheek; 5. Piriform fossa; 6. Lateral chin

During preparation, topical anesthetic was applied to the participants’ facial injected region. The calcium hydroxyapatite fillers injection (CaHA, Radiesse® plus) also contains integral 0.3% lidocaine. The modified six injection points were identified and injected with filler boluses in the supraperiosteal plane using the following protocol (Figure 1 and Table 1):

- *Eyebrow arch*, 0.3mL of CaHA, using a 25-gauge needle (25 mm).
- *Lateral eyebrow, lateral zygoma,* and *anteromedial cheek*, 0.2–0.3mL of CaHA, using a 25-gauge needle (25 mm)
- *Piriform fossa* and *the chin*, 0.2–0.3 mL of CaHA with a 25-gauge needle (23 mm).

**Table 1:** Injection plan

Point*	Description	Injection volume (ml)
1	Eyebrow arch	0.25
2	Lateral eyebrow	0.25
3	Lateral zygoma	0.25
4	Anteromedial cheek	0.25
5	Piriform fossa	0.25
6	Lateral chin	0.25

^*^Corresponding to the point illustration of Figure 1.

The entire procedure was conducted by the same physician, and the bolus injections were then repeated on the collateral side. Each participant received 12 points in total. One treatment session lasted 10–15 minutes. All patients received 3.6 mL of filler.

Standardized advanced imaging photographs (OBSERV A+, Myguard, Taoyuan, Taiwan) of the frontal (0°) were obtained pre-treatment and on day 60 and 90 post-treatment.

We assessed efficacy at week 0 (immediately post-treatment) and at weeks 4, 12, and 24 using our specialized angular and length measurement system, as well as advanced anti-aging scales.

### Anti-aging Scale System

In our previous studies, we developed a specific assessment system to evaluate the efficacy of facial rejuvenation using four angular and six linear measurements [7, 8, 10]. Based on prior experience, the minimum and maximum post-procedure deviation values were established for each of the 10 measurements. The deviation range was divided into 11 equal categories, with scores ranging from 0 to 10. The midpoint of the scale, 5, corresponds to zero deviation, representing “no change” or “neutrality”. Scores from 0 to 4 indicate aging, while scores from 6 to 10 reflect rejuvenation.

The four angles evaluated in our study are *Eyebrow-Peak, Eyebrow-Tail, Pupil-Eyebrow Peak, and Canthus-Oral-Nasal*. Following the procedure, the minimum and maximum deviation values for each angle range from −10° to +10°. A deviation between −1.11° and +1.11° post-treatment indicates no significant change. In such cases, the respective angle is assigned a neutral score of 5.

To assess the aging of periocular tissues, four additional linear measurements are evaluated: *Eyebrow-Orbital, Orbital–Upper Eyelid, Vertical–Palpebral–Fissure, and Eyebrow–Iris lengths*. These dimensions typically change subtly over time. The minimum and maximum deviation values are set at −3 mm and +3 mm, respectively. When post-treatment measurements exceed these limits (>3 mm), a score of 10, indicating supreme rejuvenation, is assigned. Conversely, extreme deviations in the opposite direction result in a score of 0. We divided the maximal amounts of deviation equally into 11 categories following the sequences (0–10). The remaining two linear lengths, *Tragus–Oral and Lower Facial Contouring lengths*, indicate the aging of the lower face. The minimum and maximum deviation values post-procedures range from −10 mm to +10 mm, the midpoint of this scale is −1.11 to +1.11mm, suggesting no changes post-treatment (Score 5). We describe the anti-aging scale briefly in the submitted table (Tables S1 and S2). We integrate the 10 measurements into precisely summative anti-aging scales. The entire anti-aging scales range from 0 to 100.

### Statistical Analyses

We used GraphPad Prism (version 6, Boston, Massachusetts, USA) for the statistical analyses. Statistical comparisons of data collected before and after the treatment were made using repeated measures ANOVA. Post hoc comparisons were made utilizing Tukey’s HSD method. We presented data as mean ± standard deviation; adjusted *p*-values less than 0.05 were deemed statistically significant. We took each side of the face as a separate unit for each patient.

## Results

**Fig. 2:**
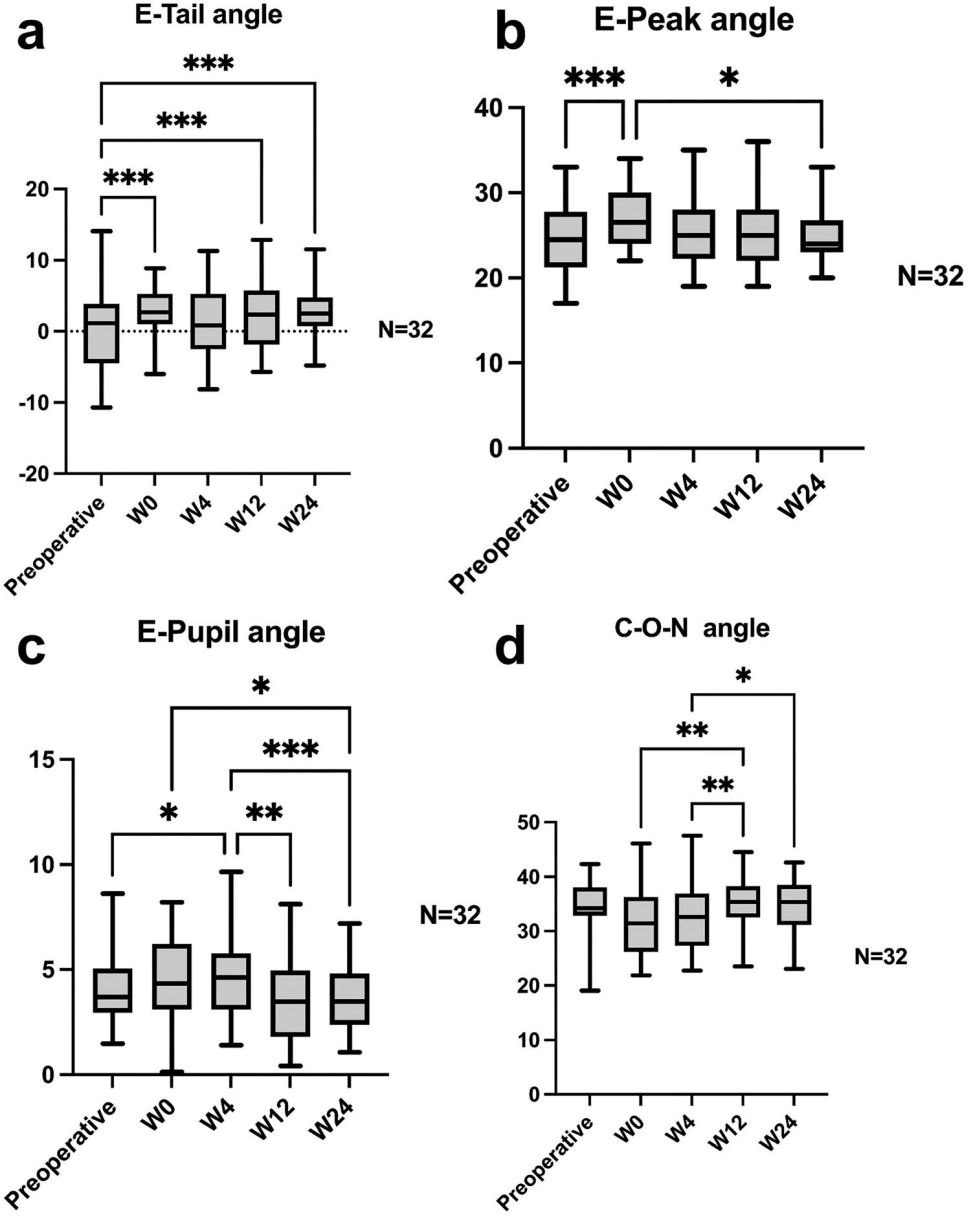
Angular measurements. **a** Eyebrow–Tail angle increased from −0.19° at baseline to 2.4° at week 12 (*p* < 0.001) and further to 2.65° at week 24 (*p* < 0.001); **b** Eyebrow Peak angle increased from 24.5° at baseline to 26.97° postoperatively (*p* < 0.001) and to 25.09° at week 24 (*p* = 0.844); **c** Pupil-Eyebrow Peak angle increased from 3.9° to 4.62° at week 4 (*p* = 0.023); this angle decreased to 3.59° at week 24 (*p* = 0.784); **d** Canthus-Oral-Nasal angle increased from 34.15° to 34.64° at week 24 (*p* = 0.96)

**Fig. 3:**
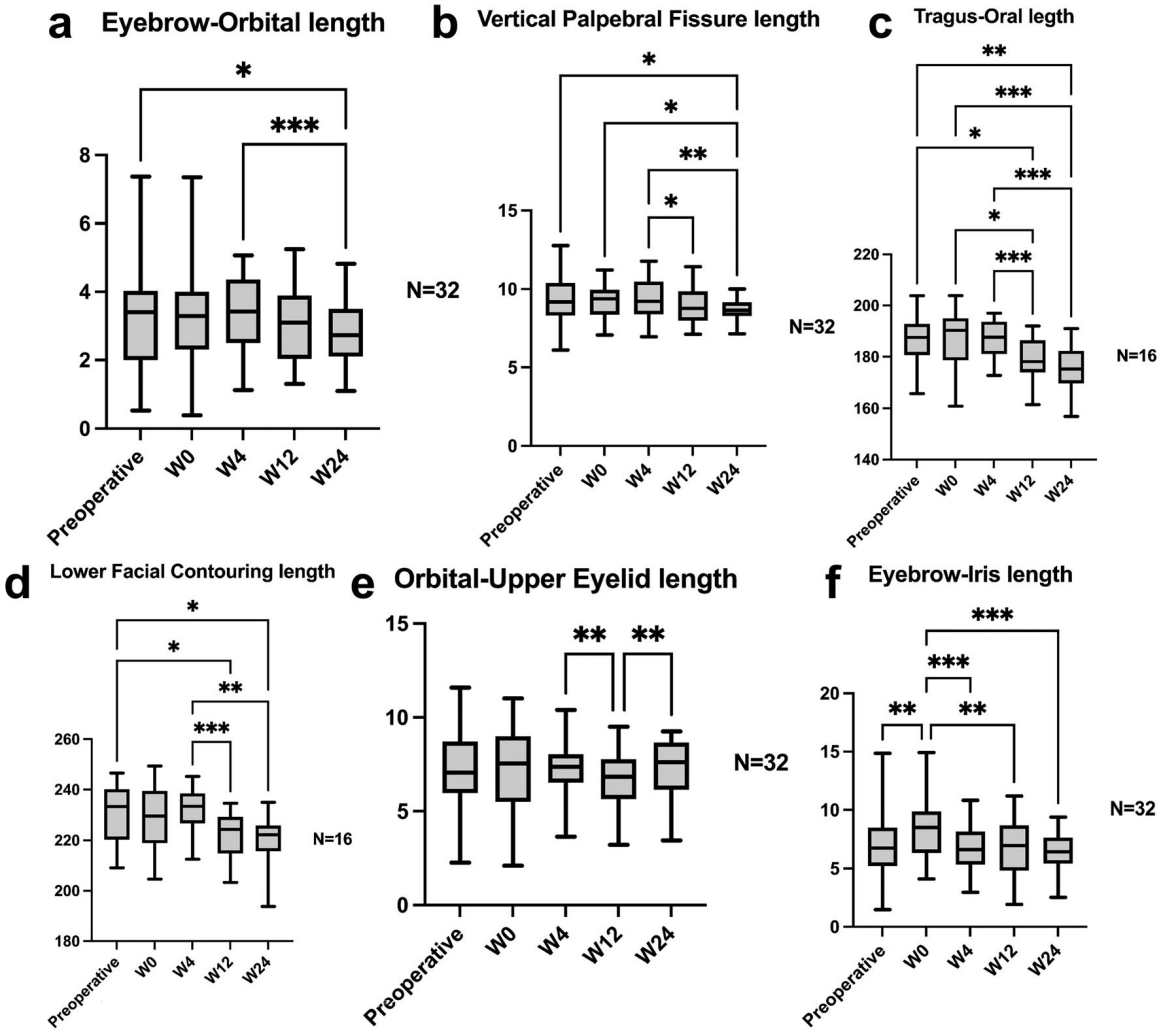
Linear measurements. The Tragus–Oral length was measured from one side of the tragus, passing through the oral commissure, to the contralateral tragus. Similarly, the Lower Facial Contouring length was measured using the same start and end points but passed through the mental protuberance instead. Consequently, the number of samples for these measurements was 16 instead of 32. **a** Eyebrow-Orbital length decreased from 3.37 to 2.76 mm at week 24 (*p* = 0.04); **b** Vertical Palpebral Fissure length decreased from 9.26 to 8.7 mm at week 24 (*p* = 0.029); **c** Tragus–Oral length decreased from 186.13 to 177.9 mm at week 12 (*p* = 0.012) and further to 175.73 mm at week 24 (*p* = 0.004); **d** Lower Facial Contouring length decreased from 230.58 to 221.93 mm at week 12 (*p* = 0.021) and further to 220.78 mm at week 24 (*p* = 0.044); **e** Orbital-Upper Eyelid length increased from 7.11 to 7.33 mm at week 24 (*p* = 0.965); **f** Eyebrow-Iris length increased from 7.03 to 8.34 mm postoperatively (*p* = 0.002); this length decreased to 6.49 mm at week 24 (*p* = 0.629)

We presented measurements from our study in Tables 2 and S3. Among the four angular and six linear parameters assessed, statistically significant variations were observed across five time points (Figures 2 and 3).

**Table 2:** Measurements at baseline (Week 0) and the end of follow-up (Week 24)

	pre-OP	W24
E-Peak angle (°)	24.50 ± 4.09	25.09 ± 3.34
E-Tail angle (°)	−0.19 ± 5.31	2.65 ± 3.59 ***
E-Pupil angle (°)	3.90 ± 1.58	3.59 ± 1.61
C-O-N angle (°)	34.15 ± 5.72	34.64 ± 5.22
E-Orbital length (mm)	3.37 ± 1.53	2.76 ± 0.85 *
Orbital-Upper Eyelid length (mm)	7.11 ± 2.20	7.33 ± 1.56
Vertical Palpebral Fissure length (mm)	9.26 ± 1.41	8.70 ± 0.65 *
E-Iris length (mm)	7.03 ± 2.62	6.49 ± 1.59
Tragus-Oral length (mm)	186.13 ± 9.23	175.73 ± 8.51 **
Lower Facial Contouring length (mm)	230.58 ± 11.81	220.78 ± 9.37 *

^*^, <0.05; **, <0.01; ***, <0.001.

Multiple comparisons were made in relation to pre-OP measurements.

*pre-OP* before the 6-point injection; *W0* immediate assessment after the 6-point injection; °: degree; mm: millimeter; *E-Peak* Eyebrow-Peak; *E-Tail* Eyebrow-Tail; *E-Pupil* Pupil-Eyebrow Peak; *C-O-N* Canthus-Oral-Nasal; *E-Orbital* Eyebrow-Orbital; *E-Iris* Eyebrow-Iris.

Regarding angular changes, the Eyebrow-Tail angle increased from −0.19 ± 5.31° at baseline to 2.4 ± 4.6° at week 12 (*p* < 0.001) and further to 2.65 ± 3.59° at week 24 (*p* < 0.001) (Figure 2a). The change of 2.84° by week 24 corresponded to an anti-aging scale score of 6, indicating eyebrow lifting. Meanwhile, the Eyebrow-Peak, Pupil-Eyebrow Peak, and Canthus-Oral-Nasal angles exhibited minimal changes by week 24, with values of 0.59° (*p* = 0.844), −0.31° (*p* = 0.784), and 0.49° (*p* = 0.96), respectively (Figure 2b, c, d). These three parameters were assigned an anti-aging scale score of 5.

Among the six measured lengths, four demonstrated statistically significant reductions by week 24. The Eyebrow-Orbital length decreased from 3.37 ± 1.53 mm to 2.76 ± 0.85 mm (*p* = 0.04), while the Vertical Palpebral Fissure length declined from 9.26 ± 1.41 mm to 8.7 ± 0.65 mm (*p* = 0.029). These reductions of −0.61 mm and −0.55 mm, respectively, resulted in anti-aging scale scores of 4 (Figure 3a and b). Additionally, the Tragus–Oral length decreased from 186.13 ± 9.23 mm to 177.9 ± 9.13 mm at week 12 (*p* = 0.012) and further to 175.73 ± 8.51 mm at week 24 (*p* = 0.004). Similarly, the Lower Facial Contouring length decreased from 230.58 ± 11.81 mm to 221.93 ± 9.12 mm at week 12 (*p* = 0.021) and to 220.78 ± 9.37 mm at week 24 (*p* = 0.044) (Figure 3c and d). These two parameters indicated recontoured the lower face. The reductions of −10.4 mm and −9.8 mm in these two parameters were associated with anti-aging scale scores of 10 and 9, respectively. The remaining two parameters, Orbital–Upper Eyelid and Eyebrow–Iris lengths, showed non-significant changes of 0.22 mm (*p* = 0.965) and −0.53 mm (*p* = 0.629) by week 24 (Figure 3e and f). Their corresponding anti-aging scale scores were 5 and 6, respectively.

**Fig. 4:**
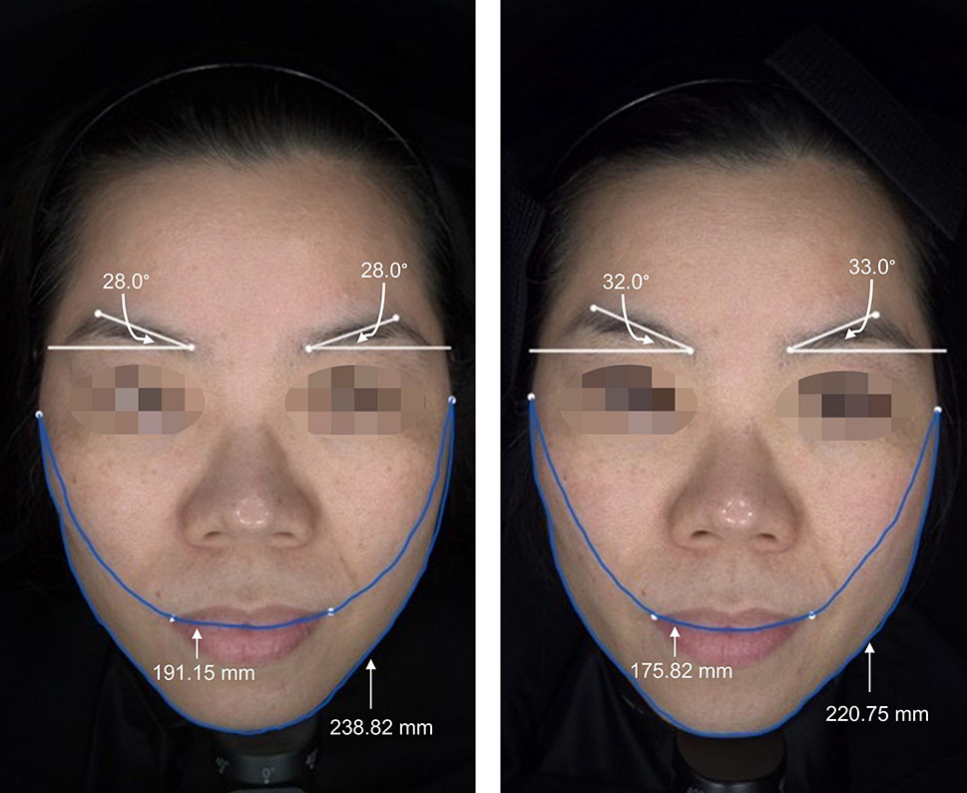
The pre-treatment appearance (left) of a female patient is shown. The post-treatment appearance (right) following one session of the 6-point injection is presented at the 24-week follow-up. The following measurements were recorded: the Eyebrow-Peak angle increased from 28.0° to 32.0° (right side) and 28.0° to 33.0° (left side); the Tragus-Oral lengths decreased from 191.15 to 175.82 mm; the Lower Facial Contouring length decreased from 238.82 to 220.75 mm. The Tragus-Oral lengths and Lower Facial Contouring length showed quantitative improvements in jawline redefining

**Fig. 5:**
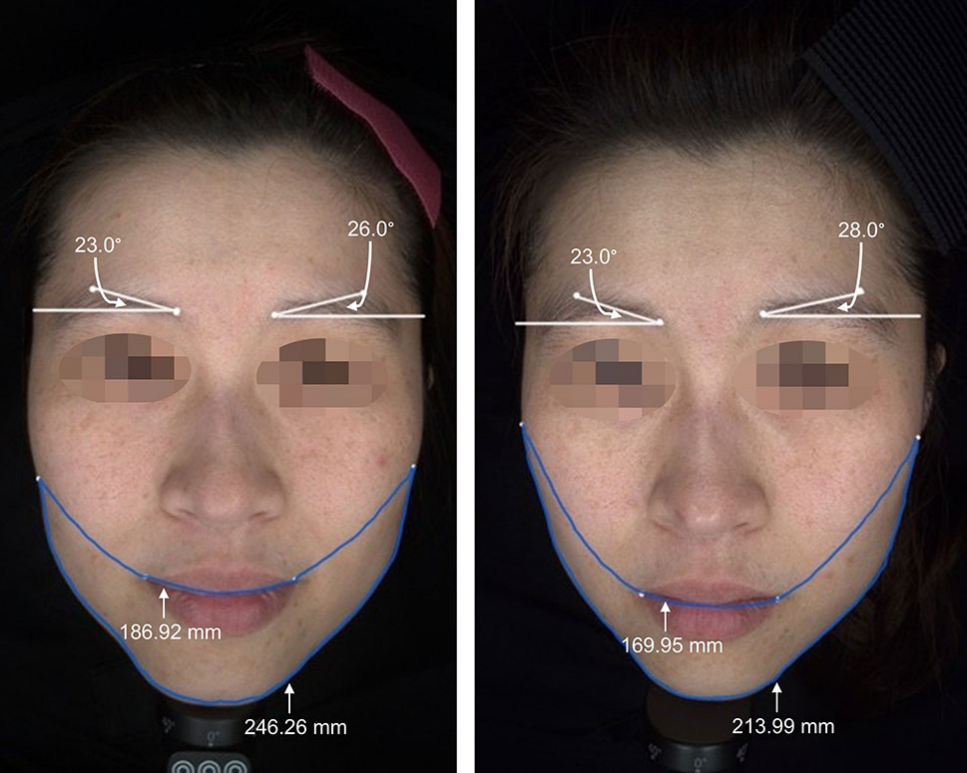
The pre-treatment appearance (left) of a female patient is shown. The post-treatment appearance (right) following one session of the 6-point injection is presented at the 24-week follow-up. The following measurements were recorded: the Eyebrow-Peak angle remained at 23.0° (right side), and this angle on the left side increased from 26.0° to 28.0°; the Tragus-Oral lengths decreased from 186.92 to 169.95 mm; the Lower Facial Contouring length decreased from 246.26 to 213.99 mm. The Tragus-Oral lengths and Lower Facial Contouring length showed quantitative improvements in jawline redefining

According to the measurements above, the final anti-aging scale score improved from an average of 50–59 by week 24, with no obvious adverse effects reported. We presented two of our cases in Figures 4 and 5.

## Discussion

Our modified 6-point approach exclusively employs the supraperiosteal plane, distinguishing it from previous studies. This refinement simplifies the procedure and enhances its accessibility for training purposes. Amaral et al. introduced the V-lift technique, a three-dimensional pan-facial treatment that employs ligament support and facial vectoring to achieve a lifting effect and restore facial contours [11]. This approach closely aligns with our concept, emphasizing the repositioning of overall facial anatomy without requiring significant volumetric augmentation. Notably, their injection technique encompasses the subcutaneous and supraperiosteal (sub-SMAS) layers. Another technique, termed the “String of Pearls”, was introduced by Sato et al [12]. This method involves two steps: first, Radiesse is injected at the palpebral-malar groove’s transition to the zygoma’s anteroinferior border, using a single entry point below the zygoma’s apex. Then, microboluses of Radiesse are retroinjected along the zygomatic area through the same entry point. The String of Pearls technique aims to enhance tissue laxity and reposition midfacial soft tissue with minimal volumetric augmentation, using a total of 0.8 mL of Radiesse distributed throughout the zygomatic area. In contrast, our technique employed a reduced volume of Radiesse, specifically 0.5 mL, targeting similar anatomical sites (lateral zygoma and anteromedial cheek) rather than utilizing a fanning technique.

When examining the effects on the forehead and periorbital area, only one parameter, the Eyebrow-Tail angle, indicated improvement. The upper periorbital region includes the eyebrows and upper eyelids, bordered by the forehead and temples. These components are interdependent and should be evaluated and treated as a unit [13]. Although the effects in periorbital area are subtle, the injection of Radiesse into the eyebrow arch and lateral eyebrow area can achieve a lifting effect comparable to that attained through the surgical procedures described by Minelli et al [14]. A subperiosteal pocket is elevated with a narrow curved periosteal dissector, and the hydroxyapatite mixture is placed along the superior orbital rim. We believe that our technique can enhance the availability of facial rejuvenation, as our 6-point injection can be performed as an outpatient procedure, in contrast to previous techniques that require general anesthesia.

Radiesse is a biodegradable and biostimulatory dermal filler that offers immediate volumetric enhancement while concurrently restoring bony contours through the stimulation of endogenous collagen production as the filler gradually degrades. The duration of its effects is long-lasting and may vary among individuals [6]. Due to its high viscosity and cohesiveness, the supraperiosteum is considered an optimal site for injection [2, 15]. In our study, Radiesse provided clinically meaningful and long-lasting improvements in jawline contour, which was also demonstrated in Green’s study [3]. Patients tolerated it well for up to 60 weeks, as assessed using the Merz Jawline Assessment Scale (MJAS) and the Subject Global Aesthetic Improvement Scale (SGAIS). Another study by Moradi et al. reported a total of 67.3% patients who responded to Radiesse jawline treatment 12 weeks after initial injection also demonstrated persistent improvement 48 weeks after initial treatment [16]. Of note, these studies evaluate the effects of jawline contouring using MJAS.

The novelty of our research lies in adopting a more scientific approach through an objective assessment system specifically developed to assess the efficacy of facial rejuvenation [7]. This reliable and accessible approach enables us to share our findings with other clinicians and patients effectively. Our measurement methods are based on quantitative criteria, which help ensure that the assessment of changes is as objective as possible [7]. Moreover, some of the changes in the defined angles and lengths were subtle, given that there are currently few articles discussing quantitative and standardized measurement methods, this is precisely the advantage and distinguishing feature of our approach. We have applied these assessments in treatments using hyaluronic acid and high-intensity focused ultrasound in previous research, demonstrating the value of our approach for evaluating various aesthetic medical procedures [9, 10].

Currently, several guidelines for the use of CaHA exist, primarily tailored to non-Asian patients [17, 18]. de Almeida et al. introduced retroinjection techniques utilizing cannulas, incorporating fanning or “asterisk” patterns with 2–4 entry points per hemiface [17]. Additionally, they proposed the short linear threading technique with needles. Similar approaches were described by Goldie et al., who also emphasized that injections should target the deep dermal or subdermal planes [18]. The pan-Asian consensus on CaHA usage strongly recommends inserting cannulas into the subcutaneous plane and administering struts or boluses along the jawline bone for cheek and midface contouring except for the zygomatic arch [6]. The modified 6-point injection aligns with 75% of attending experts who advocate for injecting filler supraperiosteally using a needle at multiple sites along the zygomatic arch. Our study builds on this clinical experience and emphasizes that Radiesse should be injected into the supraperiosteal plane, located beneath the musculoaponeurotic layer, to achieve optimal lifting outcomes [19].

The facial contours of Asian populations differ significantly from those of Western populations. Asian faces often have a flatter and broader appearance with prominent zygomatic arches and mandible angles, contributing to a rounder facial contour. In contrast, Western populations typically exhibit a more angular and narrower facial structure [20–22]. Additionally, individuals of Asian descent tend to have a wider intercanthal distance and a broader nasal base than Caucasians [23]. This discrepancy may be illustrated through the aging process. The aging process in Asian populations involves continuous changes in the midfacial skeleton, with significant decreases in certain midfacial angles over time. These changes differ from those observed in Caucasian populations, suggesting that the aging process affects facial structures differently across ethnicities [24, 25]. From an aesthetic perspective, Asian females showed a pronounced preference for a narrow lower face characterized by either a pointed or rounded chin, while a square lower face was the least preferred [26]. Our modified 6-point injection technique is more suitable for facial rejuvenation in Asian individuals, reflecting the importance of achieving balanced and natural-looking beautification across all age groups within this demographic [27].

In the pursuit of a reliable and readily accessible method for quantifying subtle changes in facial characteristics, we present four angular and six linear measurements between static and dynamic points [7]. These measurements are designed to assess minor alterations before and after rejuvenation procedures, thereby facilitating quantifying facial rejuvenation about aging. In addition to demonstrating treatment efficacy among clinical practitioners, we contend that a well-designed yet easily communicable instrument, which includes informative contexts, is a crucial factor in clinical practice. We have categorized the anti-aging scale based on each proposed measurement. In contrast to previous assessments limited to localized landmarks or regions, our scales offer an objective, precise, and comprehensive evaluation of the facial features post-treatment [28, 29].

Several limitations should be acknowledged. Firstly, the maximum follow-up interval was 24 weeks post-injection. Extended follow-up periods are necessary to validate the sustained effects of the techniques employed. Secondly, although our results demonstrate objective findings, subjective satisfaction remains equally important, as evaluations by both physicians and patients are inherently subjective. Moreover, subtle changes in the defined angles and lengths did exist, and the random variability could not be minimized due to the small sample size. Additionally, it is plausible that the lifting effects could be enhanced by combining our techniques with other aesthetic procedures, as the pan-Asian consensus suggests that lifting injections could be combined with same-day microfocused ultrasound with visualization (MFU-V). However, this hypothesis needs to be validated in further clinical trials.

## Conclusions

Our six-point calcium hydroxyapatite (CaHA) filler injection significantly achieved jawline refining and lifted the eyebrow after one session. Our specialized angular and length measurement system also provided comprehensive and informative results, facilitating effective communication between patients and physicians.

## Supplementary Information

Below is the link to the electronic supplementary material.

Supplementary file1 (DOCX 25 KB)

## Data Availability

All data included in this study are available by contacting the corresponding author upon reasonable request.
