# Supplementary material for: A Novel Six-Point Supraperiosteum Injection with Calcium Hydroxyapatite for Jawline Refining and Facial Anti-aging in Asian Patients
Source: Aesthetic Plast Surg. 2025 Jul 3;49(19):5316–24. doi: 10.1007/s00266-025-05045-x (PMC12594742; doi:10.1007/s00266-025-05045-x)
Supplement: Supplementary file 1 — Supplementary file1 (DOCX 25 KB) [file 266_2025_5045_MOESM1_ESM.docx]

| **Table S1. Movement of the Linear and Angular Assessments When Rejuvenation Present After Treatment** | |
| --- | --- |
| Length | Movement When Rejuvenation Present |
| Eyebrow-Orbital length | ↑ increased |
| Orbital-Upper Eyelid length | ↓ decreased |
| Vertical Palpebral Fissure length | ↑ increased |
| Eyebrow-Iris length | ↓ decreased |
| Tragus-Oral length | ↓ decreased |
| Eyebrow Peak angle | ↑ increased |
| Eyebrow Tail angle | ↑ increased |
| Pupil-Eyebrow Peak angle | ↓ decreased |
| Canthus-Oral-Nasal angle | ↑ increased |

| **Table S2. Table that shows the antiaging scale system** | | | | | |
| --- | --- | --- | --- | --- | --- |
| Angular | | | Linear | | |
| Index | Eyebrow-Peak/  Eyebrow-Tail/  Canthus-Oral-Nasal angle | Pupil-Eyebrow Peak angle | Eyebrow-Orbital/  Vertical-Palpebral-Fissure length | Orbital-Upper Eyelid/  Eyebrow-Iris length | Tragus-Oral/  Lower Facial Contouring length |
| 0 | < -10.00° | > 10.00° | < -3mm | > 3mm | > 10mm |
| 1 | -7.79° to -10.00° | 7.79° to 10.00° | -2.34 to -3.00mm | 2.34 to 3.00mm | 7.79 to 10.00mm |
| 2 | -5.57° to -7.78° | 5.57° to 7.78° | -1.68 to -2.33mm | 1.68 to 2.33mm | 5.57 to 7.78mm |
| 3 | -3.34° to -5.56° | 3.34° to 5.56° | -1.01 to -1.67mm | 1.01 to 1.67mm | 3.34 to 5.56mm |
| 4 | -1.12° to -3.33° | 1.12° to 3.33° | -0.34 to -1.00mm | 0.34 to 1.00mm | 1.12 to 3.33mm |
| 5 (no changes) | -1.11° to +1.11° | -1.11° to +1.11° | -0.33 to +0.33mm | -0.33 to +0.33mm | -1.11 to +1.11mm |
| 6 | 1.12° to 3.33° | -1.12° to -3.33° | 0.34 to 1.00mm | -0.34 to -1.00mm | -1.12 to -3.33mm |
| 7 | 3.34° to 5.56° | -3.34° to -5.56° | 1.01 to 1.67mm | -1.01 to -1.67mm | -3.34 to -5.56mm |
| 8 | 5.57° to 7.78° | -5.57° to -7.78° | 1.68 to 2.33mm | -1.68 to -2.33mm | -5.57 to -7.78mm |
| 9 | 7.79° to 10.00° | -7.79° to -10.00° | 2.34 to 3.00mm | -2.34 to -3.00mm | -7.79 to -10.00mm |
| 10 | > 10° | < -10.00° | > 3mm | < -3mm | < -10.00mm |
| Index 5 is identified as “neutral” or no significant changes”. Total score for the whole face was 100; either lower or higher than 50 suggesting “aging” or “rejuvenation,” respectively. °: degree; mm: millimeter. | | | | | |

| **Table S3. Detailed measurements of this study** | | | | | | | | | |
| --- | --- | --- | --- | --- | --- | --- | --- | --- | --- |
|  | pre-OP | W0 | *p*-value | W4 | *p*-value | W12 | *p*-value | W24 | *p*-value |
| E-Peak angle (°) | 24.50 ± 4.09 | 26.97 ± 3.57 | <0.001 *** | 25.69 ± 4.07 | 0.422 | 25.16 ± 3.67 | 0.871 | 25.09 ± 3.34 | 0.844 |
| E-Tail angle (°) | -0.19 ± 5.31 | 2.48 ± 4.0 | <0.001 *** | 1.20 ± 4.92 | 0.284 | 2.4 ± 4.60 | <0.001 *** | 2.65 ± 3.59 | <0.001 *** |
| E-Pupil angle (°) | 3.90 ± 1.58 | 4.52 ± 1.93 | 0.249 | 4.62 ± 1.87 | 0.023 * | 3.65 ± 1.94 | 0.820 | 3.59 ± 1.61 | 0.784 |
| C-O-N angle (°) | 34.15 ± 5.72 | 32.18 ± 6.09 | 0.111 | 32.24 ± 6.04 | 0.116 | 34.98 ± 4.88 | 0.753 | 34.64 ± 5.22 | 0.960 |
| E-Orbital length (mm) | 3.37 ± 1.53 | 3.31 ± 1.39 | 0.989 | 3.42 ± 1.10 | >0.999 | 3.02 ± 1.09 | 0.345 | 2.76 ± 0.85 | 0.040 * |
| Orbital-Upper Eyelid length (mm) | 7.11 ± 2.20 | 7.10 ± 2.18 | >0.999 | 7.15 ± 1.42 | >0.999 | 6.67 ± 1.54 | 0.483 | 7.33 ± 1.56 | 0.965 |
| Vertical Palpebral Fissure length (mm) | 9.26 ± 1.41 | 9.16 ± 1.08 | 0.991 | 9.44 ± 1.27 | 0.786 | 8.99 ± 1.15 | 0.488 | 8.70 ± 0.65 | 0.029 * |
| E-Iris length (mm) | 7.03 ± 2.62 | 8.34 ± 2.32 | 0.002 ** | 6.61 ± 1.99 | 0.765 | 6.81 ± 2.53 | 0.991 | 6.49 ± 1.59 | 0.629 |
| Tragus-Oral legth (mm) | 186.13 ± 9.23 | 187.16 ± 11.27 | 0.978 | 187.20 ± 6.98 | 0.967 | 177.90 ± 9.13 | 0.012 * | 175.73 ± 8.51 | 0.004 ** |
| Lower Facial Contouring length (mm) | 230.58 ± 11.81 | 229.31 ± 13.72 | 0.988 | 232.25 ± 8.55 | 0.951 | 221.93 ± 9.12 | 0.021 * | 220.78 ± 9.37 | 0.044 * |
| *, <0.05; **, <0.01; ***, <0.001. Multiple comparisons were conducted in relation to pre-OP measurements. pre-OP, before the 6-point injection; W0, immediate assessment after the 6-point injection; °: degree; mm: millimeter; E-Peak, Eyebrow–Peak; E-Tail, Eyebrow–Tail; E-Pupil, Pupil–Eyebrow Peak; C-O-N, Canthus–Oral–Nasal; E-Orbital, Eyebrow-Orbital; E-Iris, Eyebrow-Iris. | | | | | | | | | |
